# Monitoring cow comfort and rumen health indices in a cubicle-housed herd with an automatic milking system: a repeated measures approach

**DOI:** 10.1186/s13620-015-0040-7

**Published:** 2015-06-12

**Authors:** Arne Vanhoudt, Steven van Winden, John C. Fishwick, Nicholas J. Bell

**Affiliations:** Department of Farm Animal Health, Utrecht University, Veterinary Faculty, Yalelaan 7, 3584CL Utrecht, The Netherlands; The Royal Veterinary College, Department of Production and Population Health, Hawkshead Lane, Hatfield, Hertfordshire AL9 7TA UK

**Keywords:** Automatic milking system, Behaviour, Cows, Cow comfort, Dairy, Rumen health

## Abstract

**Background:**

Cow rumination and lying behaviour are potentially useful and interrelated indicators of cow health and welfare but there is conflicting evidence about how reliable these measures are. The objective of this study was to quantify the variation of indices of cow comfort and rumen health in a herd with an automatic milking system for which husbandry was relatively constant, in order to propose an alternative approach to optimising the use of these indices when continuous monitoring is not available. During a period of 28 days, standing index, cud chewing index and rumination index were observed.

**Results:**

The daily mean standing index ranged between 9.0 and 18.0 per cent, cud chewing index between 43.5 and 74.0 per cent, and rumination index between 49.0 and 81.0 per cent. The point of lowest variation in the indices was determined as that with the lowest coefficient of variation. The coefficient of variation was lowest for data collected between 240 and 270 minutes after refreshing of the bedding material on the cubicles for both the standing index and rumination index, and for data collected between 120 and 150 minutes after refreshing of the bedding material on the cubicles for the cud chewing index.

**Conclusions:**

In spite of relative constant husbandry practices in a herd with an automatic milking system, the variation in the standing index, cud chewing index and rumination index was still considerable. This suggests these measures should be repeated on several consecutive days, according to population size and wanted margin of error, to be representative and useful.

## Background

Resting behaviour and rumination are a prerequisite and indicator of good cow health and welfare. Consequently, ways of evaluating and monitoring these have been an active area of research in recent years. The standing index (SI), cud chewing index (CudCI) and rumination index (RI) are three measures that have been considered for the monitoring of animal welfare, production and disease [1–3]. Evaluation of indices of cow comfort and rumen health forms an important part of welfare assessment protocols and herd health evaluations on dairy farms.

Numerous approaches have been investigated for assessing resting behaviour but few found to be reliable and feasible to be assessed on a single time-point [4]. Standing index, sometimes referred to as stall standing index, measures the proportion of cows touching a cubicle that are not lying down in it [5]. The same authors describe the SI as the inverse of the cow comfort index (CCI) or cow comfort quotient which measures the proportion of cows touching a cubicle that are actually lying down (SI = 1 - CCI). Overton et al. [6] have suggested that a CCI greater than 85 per cent at approximately one hour after the cows return from the early morning milking is to be considered the desired goal, whilst Cook et al. [1] suggested that a SI greater than 20 per cent at two hours before the morning or afternoon milking is indicative of excessive herd mean stall standing times. Several studies have used the CCI or SI or both as animal-based measures of cow comfort [1, 7, 8].

Farms with optimal rumen health are more likely to be profitable due to efficient nutrient utilisation [9]. Furthermore, increased rumination is generally associated with an improved health status and is positively correlated with milk yield [2]. Cows have been found to spend between 5 and 10 hours per day ruminating [10, 11, 2], with stress, anxiety, disease and discomfort all resulting in decreased rumination [12, 13, 2]. Consequently, rumination monitoring can be used to evaluate the health status and cow comfort of a herd [7, 2].

Rumination has been studied at herd level by monitoring the CudCI, defined as the number of cows that were lying down in cubicles and ruminating multiplied by 100 divided by the total number of cows lying down in the cubicles [7]. Alternatively, the proportion of cows in the herd that are ruminating at any given time can be assessed. At least 40 per cent of the cows in a herd ruminating at any given time has been suggested to reflect good rumen health [11, 14, 15].

During the past decade, automatic milking systems (AMS) have been more widely adopted by dairy producers. Despite this increase in use and popularity, to the best knowledge of the authors, no papers have been published before now on the indices of rumen health in herds with an AMS and only one with data on the CCI in herds with an AMS [8]. The latter paper reported a CCI of 80.0 per cent ± 6.8 (mean ± se) from five Dutch herds with an AMS, with four observations within one day (9 h30, 11 h30, 13 h30 and 15 h30) for each farm [8].

Currently when assessing the level of cow comfort, health and welfare of cattle on dairy farms in the absence of continuous monitoring, most advisers and managers have to rely on formal or informal behavioural observations such as SI, CudCI and RI. There is some evidence to validate these measures at a single time-point; no studies have examined approaches to establish the time-point with lowest variation due to extraneous factors other than those being evaluated. This paper presents a more robust statistical approach, the calculation of the coefficient of variation, for establishing the time-point with lowest variation in these indices for a herd managed under relatively constant conditions and milked with an AMS. Furthermore, the precision of an observed mean can be increased by increasing the number of observations, namely a repeated measures approach.

The aim of this study was to quantify the variation in SI, CudCI and RI, in a herd with an AMS demonstrating signs of normal rumen health. The authors propose an alternative approach for determining the optimal times and number of repeated observations for assessing these indices in herds with an AMS in order to minimise the influence of natural variation in the behaviours observed with the SI, CudCI and RI.

## Methods

### Ethical approval

The study was reviewed and approved on February 9, 2013 by the Royal Veterinary College Ethics and Welfare Committee under the unique reference number 2012 1196.

### Study population

A study population of pedigree Holstein cows in a single herd based in Somerset, UK, was observed between March 4 and April 15, 2013. Throughout this period, there was a median of 125 (range: 121 to 128) lactating animals. Cows were voluntarily milked through two robots (LELY ASTRONAUT *A3 Next* Milking Robot, Lely Industries N.V., The Netherlands) with free cow traffic to the AMS. Concentrate was fed in the AMS and a mixed ration in a feed passage with a total feed barrier length of 74.1 metres (at least 0.58 metre feed space per cow). Cows were continuously housed, bedded on sawdust on two year old latex foam inserts (Premium pad, Wilson Agriculture, UK) on top of 14 year old rubber-crumb filled mattresses (Pasture mats, Wilson Agriculture, UK) with European supercomfort cubicles including a bracket-raised neck rail (Dutch Comfort, De Boer Housing Systems, UK).

A subsample consisting of the most recently calved cows that appeared clinically healthy following a full clinical examination at the start of the study were recruited until a number of 35 cows was reached. A pH bolus (smaXtec pH Bolus SX-1042, smaXtec animal care GmbH, Austria) was administered and rumination collar (Heatime Vocal, HR-Tag, Fabdec, UK) fitted to this subsample. The reticuloruminal pH was recorded by the pH bolus every 10 minutes for a maximum measuring period of 50 days following activation. A mobile reader (smaXtec Mobile Reader SN-4042, smaXtec animal care GmbH, Austria) was positioned near the AMS and the recorded pH data were transmitted to the mobile reader via radio-transmission each time the pH bolus was within the proximity of 5 metres. Work by Gasteiner et al. [16] has validated the pH bolus comparing results from standardised solutions (pH 4, pH 7) with in-vivo measurements under different feeding conditions. Rumination time was recorded by the rumination collars and technical details are described by Schirmann et al. [17]. The data from the mobile reader and rumination collars were exported to a digital spreadsheet (Microsoft Excel, Microsoft, USA) for further analysis. The observer (AV) was blinded to the pH and rumination collar data during the study period.

### Observations

A methodology described by Cook et al. [1] was adapted for the purpose of this study. At the start of the study, facility design and management practices were recorded. Throughout the entire study period, the observer was present on the farm from 7 h30 until 17 h30. Each day, every hour from 8 h00 until 17 h00, each cubicle was inspected and it was noted when a cow was present, if that cow was standing or lying, and ruminating or not. A cow was defined as ruminating if she could be seen chewing cud whilst being observed for approximately 10 seconds. During this time period, interventions from the farm personnel (for example the cleaning of the cubicles or pushing-up of the feed at the feed barrier) were observed and recorded. Following the start of the study, cows were given a 15 day period of adaptation to the presence of the observer. During this adaptation period, all observations were done as during the rest of the study. The observed data were recorded on paper sheets and then entered into a digital spreadsheet (Microsoft Excel, Microsoft, USA) for calculation of the indices of cow comfort and rumen health. Data collected during the adaptation period were excluded from the data analysis. This resulted in the analysis of data recorded during the last 28 days of the study period.

The SI was defined as the number of cows touching a cubicle but not lying multiplied by 100 divided by the total number of cows touching the cubicles. The CudCI was calculated as the number of cows that were lying down in the cubicles and ruminating multiplied by 100 divided by the total number of cows lying down in the cubicles and the RI as the number of cows that were touching a cubicle and ruminating multiplied by 100 divided by the total number of cows touching the cubicles.

Relative humidity and environmental temperature were recorded every 40 seconds in a central area of the pen using a data logger (CEM DT-172, CEM, Shenzhen Everbest Machinery Industry Co., China). The temperature-humidity index (THI) was calculated according to the method described by Bohmanova et al. [18].

Data such as milking visits per day per cow, number of cows in the milking herd and intake of concentrate per visit to the AMS were captured automatically by the AMS.

The cows were denied access to the cubicles for about 30 minutes immediately after the provision of fresh feed in the morning. During this time, dung was removed from the back third of the cubicles. Subsequently, a thin layer of hydrated lime was spread on the clean cubicles, which was topped with new sawdust. Hereafter, access to the cubicles for the cows was restored. To align the hourly observations of the indices from each day, the refreshing of the bedding material on the cubicles was used as reference point (T0) rather than milking time as for conventionally milked (non-AMS) herds. Observations made after the first disturbance (for example pushing-up of the feed at the feed barrier) of the cows by the farm personnel after T0, were excluded from the data analysis.

### Statistical analysis

The data for each parameter were tested for normality with the Shapiro-Wilk test and appropriate measures of central tendency and variation were calculated using IBM SPSS Statistics 20 (IBM Limited, UK). Following alignment of the data at T0, the data were grouped in 30 minutes time-slots after T0. Each grouped 30 minutes time-slot contained data from the observations made throughout the 28 days study period. The mean centred coefficient of variation (CV) was calculated for each grouped time-slot by taking the standard deviation and dividing it by the mean, then multiplying by 100. Grouped time-slots with data for less than 50 per cent of the days were excluded from the analysis.

The precision of the observed mean can be increased by increasing the number of observations, that is repeated measures. The maximum difference between the observed mean and the population mean is defined as the margin of error (E) and is half the width of the 95 per cent confidence interval. The number of observations (n) needed to achieve a stated margin of error of the mean $\left(\overline{\mathrm{x}}\right)$ was calculated using the following formula:

$$ \mathrm{n}={\left[\frac{1.96\times sd}{E}\right]}^2={\left[\frac{1.96\times sd}{UB-\overline{x}}\right]}^2 $$

sd: standard deviation

UB: upper bound of the confidence interval

## Results

During the study period, the median number of visits to the milking robot per cow per day was three with an interquartile range (IQR) of two and the median milk yield per cow per day was 34.0 (IQR 16) kg. Cows were found to have a median intake of 2.080 (IQR 0.984) kg of concentrate per visit to the AMS with a minimum and maximum intake of 0.053 and 3.073 kg of concentrate respectively. The median intake of concentrate at the AMS per cow per day was 6.117 (IQR 4.070) kg with a minimum and maximum intake of 0.477 and 13.000 kg respectively. Stocking density, defined as the number of cows per 100 cubicles [1], ranged between 98 and 103 per cent throughout the study. The daily mean THI remained below 68 throughout the study.

Due to failure of the pH bolus, two cows were excluded from the data analysis for the subsample which was constituted by the cows with a pH bolus and rumination collar. During the study, the cows in the subsample were a median of 92 (IQR 49) days in lactation. Median milk yield per cow per day was 41.0 (IQR 14) kg. These cows had a median intake of 2.430 (IQR 1.008) kg of concentrate per visit to the AMS with a minimum and maximum intake of 0.630 and 3.073 kg of concentrate respectively. The median intake of concentrate at the AMS per cow per day was 8.242 (IQR 2.927) kg with a minimum and maximum intake of 1.802 and 13.000 kg respectively. A reticuloruminal pH lower than 5.5 was recorded in one cow from the subsample at one occasion on day 26. The period with a reticuloruminal pH below 5.5 was from 21 h16 until 22 h06 with 5.3 being the lowest pH recorded. For all cows in the subsample, all the other reticuloruminal pH recordings were at least 5.5 throughout the study. The mean daily rumination time of cows in the subsample ranged between 412 and 514 minutes (6.9 and 8.6 hours) during the study.

**Fig. 1:**
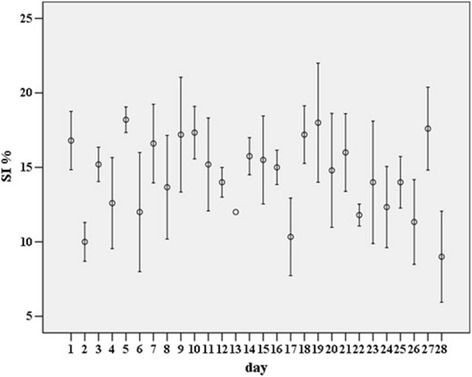
Mean standing index (SI %) ± SE on each day of the study

**Fig. 2:**
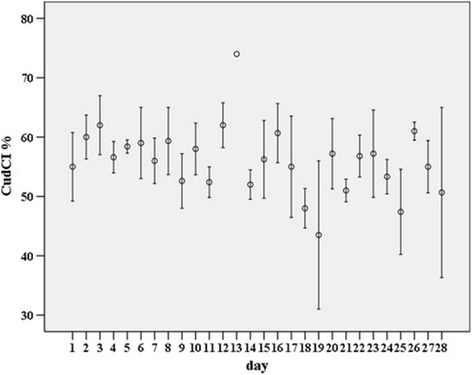
Mean cud chewing index (CudCI %) ± SE on each day of the study

**Fig. 3:**
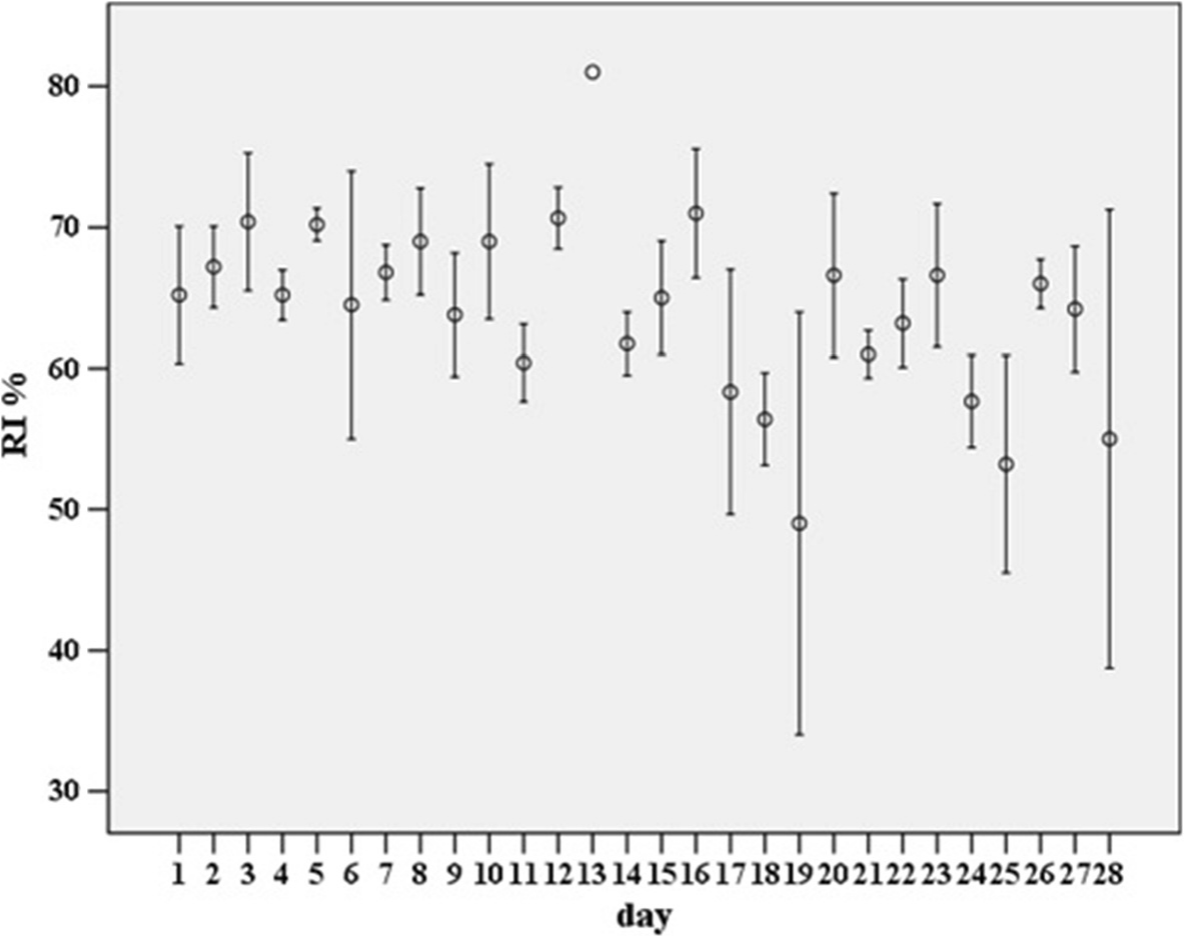
Mean rumination index (RI %) ± SE on each day of the study

Each index was calculated for a total of 112 hourly observations, with a median of five (IQR two) observations per day. The mean SI for the observations on each day, hereafter named ‘daily’, ranged between 9.0 and 18.0 per cent, daily mean CudCI between 43.5 and 74.0 per cent, and daily mean RI between 49.0 and 81.0 per cent. The difference between the mean CudCI and RI for each day was 8.1 per cent ± 0.4 (mean ± se). The within and between day variation during the observed timeframe of the SI, CudCI and RI is shown in Figs. 1, 2 and 3 respectively. For each time-slot following the refreshing of the bedding material on the cubicles, the mean, sd and CV for the SI, CudCI and RI was calculated (Table 1). The CV was lowest for data collected between 240 and 270 minutes after refreshing of the bedding material on the cubicles for both the SI (CV = 23.7) and RI (CV = 7.7) and for data collected between 120 and 150 minutes after refreshing of the bedding material on the cubicles for the CudCI (CV = 10.5).

**Table 1:** The mean (%), standard deviation (SD) and mean centred coefficient of variation (CV) for standing index, cud chewing index and rumination index in relation to time after refreshing of the bedding material on the cubicles

Minutes	N	Standing index			Cud chewing index			Rumination index		
		Mean	SD	CV	Mean	SD	CV	Mean	SD	CV
60-90	26	12.1	5.2	43.0	57.2	15.4	26.9	63.0	17.5	27.8
120-150	27	11.3	4.4	38.9	62.4	6.5	10.4	69.0	6.3	9.1
180-210	25	16.1	4.1	25.5	53.6	6.4	11.9	63.2	6.3	10.0
240-270	19	19.1	4.5	23.6	49.5	6.2	12.5	62.4	4.8	7.7
300-330	15	17.0	4.4	25.9	51.1	7.0	13.7	60.2	7.2	12.0

The number of repeated observations needed to achieve a stated margin of error was calculated using the observed values at the moment of lowest coefficient of variation for the mean and standard deviation of each index (Table 2). The number of repeated observations needed to achieve an observed mean with a margin of error of 5.0 per cent was three observations for the SI, six observations for the CudCI and four observations for the RI. To decrease the margin of error to 2.5 per cent, the number of repeated observations needed was 12, 26 and 14 for the SI, CudCI and RI respectively.

**Table 2:** The number of repeated observations needed to achieve a stated margin of error for each of the indices, at their respective point of lowest coefficient of variation

Index	Mean (%)	Margin of error (%)	Number of repeated observations	95 % confidence interval
Standing index	19.1	8.8	1	10.3 – 27.9
		5	3	14.1 – 24.1
		2.5	12	16.6 – 21.6
Cud chewing index	62.4	12.7	1	49.7 – 75.1
		5	6	57.4 – 67.4
		2.5	26	59.9 – 64.9
Rumination index	62.4	9.4	1	53.0 – 71.8
		5	4	57.4 – 67.4
		2.5	14	59.9 – 67.9

## Discussion

The SI, CudCI and RI are putative indicators of cow comfort, health and welfare. However, no studies have evaluated these parameters intensively over successive days using a population of cows with normal rumen health (monitored through measurement of reticuloruminal pH and rumination time of a subsample in the population), in absence of heat stress (THI < 68) and relatively constant day-to-day management afforded by the AMS. Furthermore, there are few reports of these indices relevant to herds milked with an AMS, which are less affected by the behavioural synchronisation that occurs with conventional milking [6, 19]. The results from this study show that the behaviours reflected by these indices can vary substantially during a day and between days on a farm with an AMS.

In contrast to herds with a conventional milking system, behavioural synchronisation in herds with an AMS does not occur with milking time as this will vary for each cow. However, a certain degree of behavioural synchronisation does occur in herds with an AMS with the provision of fresh feed and/or the cleaning and refreshing of the bedding materials of the cubicles. These moments are suitable time-points from which the moment with lowest variation in the behaviours observed with the SI, CudCI and RI can be determined.

In this study, the CV was used to determine the time-point with lowest variation due to extraneous factors other than those being evaluated. For the CudCI, this was lowest between 120 and 150 minutes after the refreshing of the bedding material on the cubicles and for both SI and RI between 240 and 270 minutes after the refreshing of the bedding material on the cubicles. A study by Schirmann et al. [20] also found that the latter time-point coincides with peak rumination following intake of fresh feed.

The SI (and CCI) has been promoted as a measure of cow comfort because it is a simple, visual assessment of cows’ interaction with a cubicle. The SI has been correlated with stall standing times in one study comparing mattress and deep sand bedding [1]. These authors concluded this index has the potential for capturing a range of cow comfort issues such as heat stress or lack of bed cushioning which are known to increase standing times. The SI of 19.1 per cent ± 1 (mean ± se; CCI = 80.9 per cent) at the moment of lowest CV from our study is in line with the findings of a study in the Netherlands that reported a CCI of 80.0 per cent ± 6.8 (mean ± se) in herds with an AMS, with four observations within one day (9 h30, 11 h30, 13 h30 and 15 h30) for each farm [8] and is lower than the stall standing index of 24 per cent ± 1.5 (least square mean ± se) at two hours before the morning or afternoon milking reported in conventionally milked herds [1]. The daily mean SI ranged between 9.0 and 18.0 per cent revealing the large variation in SI, which was notable between and within days. This supports the view that there are many factors other than environmental comfort influencing the SI. One Canadian study concluded that the CCI from a single observation two hours before milking is not correlated with daily lying times measured over 5 days [21]. This study concluded that measures such as CCI are not to be used as single point observations to assess cow lying behaviour on herds with a conventional milking system. Therefore, continuous measurement of lying time is considered best practice to evaluate cow lying behaviour.

The cows in the subsample were considered at highest risk for low reticuloruminal pH due to their yield (median 41 kg/d, IQR 14) and stage of lactation (median 92 days in lactation, IQR 49) during the observation period. Despite this higher risk, the measured reticuloruminal pH remained within normal range with the exception of one cow for approximately one hour on day 26, indicating this herd had good rumen health. Daily mean RI ranged between 49.0 and 81.0 per cent, compared with CudCI which ranged between 43.5 and 74.0 per cent. All the measures exceeded the threshold of 40 per cent consistent with good rumen health as reported by DeVries et al. [15]. This study found a difference of 8.1 per cent ± 0.4 (mean ± se) between the mean CudCI and RI of each day. This poses a question as to which is the most appropriate parameter to monitor. As the CudCI excludes cows standing in the cubicles from the calculation, it has the potential of underestimating the rumen health status of the herd when a large number of cows are standing in cubicles due to lameness, heat stress, poor cubicle comfort or variation in standing index. To avoid this, the authors suggest that in the absence of continuous measurement of the reticuloruminal pH or rumination time, the RI should be preferred over the CudCI.

The calculation of the number of repeated observations needed to achieve a margin of error of 5.0 per cent indicated that three and four repeated observations are needed for the SI and RI respectively. Further decreasing the margin of error of the observed mean to 2.5 per cent, resulted in an increased number of repeated observations to 12 and 14 for the SI and RI respectively. These results support the findings from Ito et al. [21] and DeVries et al. [15] that single observations of the SI and RI are unlikely to result in reliable measures of the behaviours reflected by these indices.

This study assessed the variability of rumination and lying (behavioural) indices in a novel way by using the point of lowest coefficient of variation and a repeated measures approach for a single herd of 125 Holsteins. However, further work could investigate this concept on a range of AMS farms with varying management conditions using the most appropriate synchronisation cues. Likewise, future work could look at the synchronisation of these behaviours in herds with conventional milking systems to establish if this methodology can also be used in these systems. Behavioural synchronisation has a large effect on welfare and clinical behaviour scoring and the return to beds after bed cleaning represented a strong cue for cows in this study.

## Conclusions

The considerable unexplained variation in SI and RI shows why a single measurement of these parameters is likely to be unreliable as found by Ito et al. [21] for CCI and DeVries et al. [15] for rumination behaviour. Continuous monitoring using lying time monitors and rumen health indicators such as measurement of reticuloruminal pH or rumination time has to remain the gold standard for these measures. However, as continuous monitoring is not available on the majority of farms at present, the authors suggest the further investigation of repeated assessment of SI and RI at the point of lowest coefficient of variation. This may improve the validity of these measures on a single farm over time. The large unexplained variation in the dataset under relatively constant conditions shows these measures have limited or no value for comparison between herds.
