# Erratum to: Monitoring cow comfort and rumen health indices in a cubicle-housed herd with an automatic milking system: a repeated measures approach

**DOI:** 10.1186/s13620-015-0046-1

**Published:** 2015-08-27

**Authors:** Arne Vanhoudt, Steven van Winden, John C. Fishwick, Nicholas J. Bell

**Affiliations:** Department of Farm Animal Health, Utrecht University, Veterinary Faculty, Yalelaan 7, Utrecht, 3584CL The Netherlands; The Royal Veterinary College, Department of Production and Population Health, Hawkshead Lane, Hatfield, AL9 7TA Hertfordshire UK

The original version of this article [1] unfortunately published with two mistakes. The PDF of the article was missing the last page. Furthermore there was a mistake in the data Table two of the original article. The corrected table is included here as Table 1. Both errors have been corrected in the original article.

**Table 1:** The number of repeated observations needed to achieve a stated margin of error for each of the indices, at their respective point of lowest coefficient of variation

Index	Mean (%)	Margin of error (%)	Number of repeated observations	95 % confidence interval
Standing index	19.1	8.8	1	10.3 – 27.9
		5	3	14.1 – 24.1
		2.5	12	16.6 – 21.6
Cud chewing index	62.4	12.7	1	49.7 – 75.1
		5	6	57.4 – 67.4
		2.5	26	59.9 – 64.9
	62.4	9.4	1	53.0 – 71.8
		5	4	57.4 – 67.4
Rumination index		2.5	14	59.9 – 64.9
